# A comparison of real-time radial GRAPPA and standard cine imaging for the evaluation of cardiac function in children and young adults

**DOI:** 10.1186/1532-429X-18-S1-O74

**Published:** 2016-01-27

**Authors:** Jooho P Kim, Jie Deng, Joshua D Robinson, Nicole Seiberlich, Cynthia K Rigsby

**Affiliations:** 1 Northwestern University Feinberg School of Medicine, Chicago, IL USA; 2 Department of Medical Imaging, Ann & Robert H. Lurie Children's Hospital of Chicago, Chicago, IL USA; 3 Department of Radiology, Northwestern University Feinberg School of Medicine, Chicago, IL USA; 4 Department of Pediatrics, Division of Pediatric Cardiology, Ann & Robert H. Lurie Children's Hospital of Chicago, Chicago, IL USA; 5 Department of Pediatrics, Northwestern University Feinberg School of Medicine, Chicago, IL USA; 6 Department of Biomedical Engineering, Case Western Reserve University, Cleveland, OH USA; 7 Department of Radiology, University Hospitals of Cleveland, Cleveland, OH USA

## Background

Segmented balanced steady state free precession (bSSFP) cine imaging is the gold standard MRI technique for assessing ventricular systolic function. The image acquisition involves ECG gating and is ideally performed during breath-hold to reduce artifacts from myocardial and respiratory motion. Real-time radial imaging with parallel imaging technique GRAPPA reconstruction (radial GRAPPA) utilizes a radial data collection scheme in combination with a through-time k-space GRAPPA technique to achieve high acceleration rates. As it is acquired during free-breathing and is not ECG-gated, it has the potential to dramatically shorten cardiac functional imaging time. As radial GRAPPA has previously only been validated in adult patients, our purpose is to compare image quality between bSSFP and radial GRAPPA for evaluating cardiac function in children and young adults.

## Methods

In this prospective, IRB-approved study, informed consent was obtained from 30 patients (median age 16.2 years, range 0.9 to 42.9 years) to undergo short axis cine cardiac imaging with bSSFP and radial GRAPPA during a clinically indicated cardiac MRI study. Both sets of images were post-processed by a single user using QMass® MR v7.6 (Leiden, Netherlands) to calculate EDV, ESV, EF, stroke volume (SV), and cardiac output (CO) for the right (RV) and left ventricles (LV). We compared these functional parameters between the two techniques using two-tailed t-tests, linear regression analyses, and Bland-Altman plots. Scan durations were compared using a two-sample t-test. Two blinded independent reviewers rated image quality using a 5-point Likert scale based on the following image features: wall motion, endocardial border, myocardium, blood pool, papillary muscles, AV valves, and presence of artifacts. Ratings were compared using a Wilcoxon rank-sum test.

## Results

Mean radial GRAPPA scan duration (4.1 ± 0.5 min) was significantly shorter than that of bSSFP (10.3 ± 4.2 min), p < 0.001. There was no significant difference between bSSFP and radial GRAPPA for LVEDV, LVESV, LVSV, LVEF, RVEDV, RVESV, or RVEF (p-values of 0.336, 0.974, 0.107, 0.117, 0.123, 0.711, and 0.137, respectively). LVCO, RVSV, and RVCO were however significantly different between the two modalities (p-values of 0.046, 0.008, and 0.033, respectively). Mean image quality ratings were significantly higher for bSSFP compared to radial GRAPPA for all features rated (p≤0.001), ranging from 4.1 to 4.8 for bSSFP and 2.4 to 3.8 for radial GRAPPA.

## Conclusions

Both bSSFP and radial GRAPPA yield similar results for most cardiac ventricular functional measurements. However, image quality ratings were lower for radial GRAPPA. Radial GRAPPA holds promise for decreasing scan times for cardiac functional analysis in children and young adults, but the parameters will need to be optimized to obtain comparable image quality for these patients.

**Table 1:** Pulse Sequence Parameters for Segmented bSSFP and Radial GRAPPA Techniques

14-15 (non breath-hold)	14-15 (non breath-hold)	14-15 (non breath-hold)
Field of view (mm^2^)	300-380 × 200-285	300-400 × 240-300
Voxel size (mm^3)	1.5-1.8 × 1.9-2.2 × 5.0-7.0	2.1-2.5 × 2.1-2.5 × 5.0-7.0
Repetition time (msec)	1.2-1.3	1.3-1.5
Echo spacing (msec)	2.5-3.0	2.6-3.1
Section thickness (mm)	5-7	5-7
Base resolution (matrix size)	160-176	128-160
Radial views	N/A	144
Flip angle (°)	90	60
Acceleration factor	iPAT = 2	Radial Grappa Acceleration R = 9-12
Bandwidth (Hz/px)	920	680-1302
Temporal resolution (msec)	21-23	31-50
Scan time (sec per slice)	10-15 (breath-hold)	14-15 (non breath-hold)

**Figure 1:**
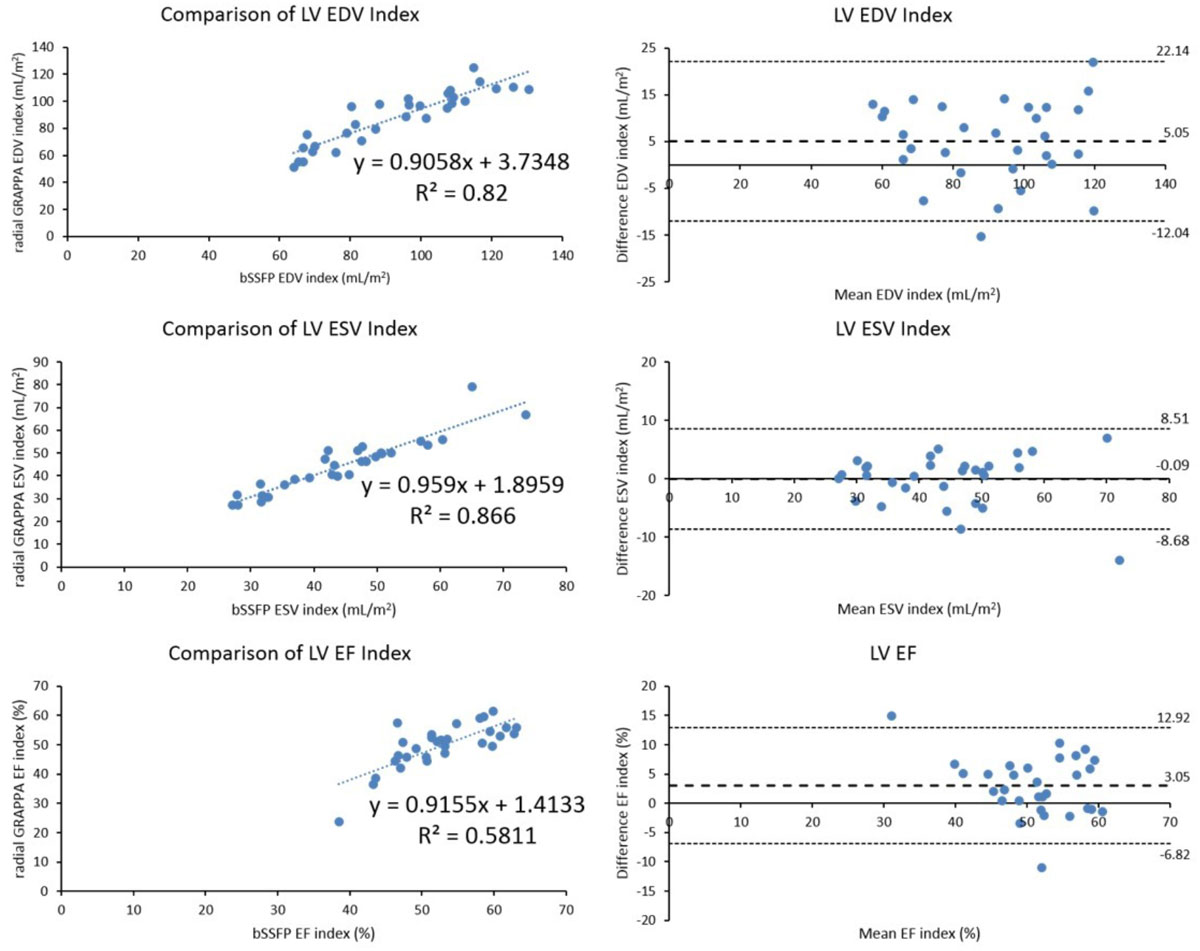
**Linear regression and Bland-Altman plots for LVEDV, LVESV, and LVSV index for all 30 patients**. The Bland-Altman plots show mean values calculated using bSSFP and radial GRAPPA techniques on the x-axis and the difference between the values calculated from each modality (bSSFP - radial GRAPPA) on the y-axis. Mean difference, as well as 95% limits of agreement, have also been plotted. Excellent correlation is seen between bSSFP and radial GRAPPA for these functional parameters.

